# Interplay of orbital effects and nanoscale strain in topological crystalline insulators

**DOI:** 10.1038/s41467-018-03887-5

**Published:** 2018-04-19

**Authors:** Daniel Walkup, Badih A. Assaf, Kane L. Scipioni, R. Sankar, Fangcheng Chou, Guoqing Chang, Hsin Lin, Ilija Zeljkovic, Vidya Madhavan

**Affiliations:** 1 Department of Physics, Boston College, Chestnut Hill, MA 02467 USA; 2 National Institute of Standards and Technology, Gaithersburg, MD 20899 USA; 3 Department of Physics and Frederick Seitz Materials Research Laboratory, University of Illinois Urbana-Champaign, Urbana, IL 61801 USA; 4 Center for Condensed Matter Sciences, National Taiwan University, Taipei, 10617 Taiwan; 5 Centre for Advanced 2D Materials and Graphene Research Centre, National University of, Singapore, 117546 Singapore; 6 Department of Physics, National University of Singapore, Singapore, 117542 Singapore; 7 Département de Physique, Ecole Normale Supérieure, PSL Research University, CNRS, Paris 75005 France

## Abstract

Orbital degrees of freedom can have pronounced effects on the fundamental properties of electrons in solids. In addition to influencing bandwidths, gaps, correlation strength and dispersion, orbital effects have been implicated in generating novel electronic and structural phases. Here we show how the orbital nature of bands can result in non-trivial effects of strain on band structure. We use scanning–tunneling microscopy to study the effects of strain on the electronic structure of a heteroepitaxial thin film of a topological crystalline insulator, SnTe. By studying the effects of uniaxial strain on the band structure we find a surprising effect where strain applied in one direction has the most pronounced influence on the band structure along the perpendicular direction. Our theoretical calculations indicate that this effect arises from the orbital nature of the conduction and valence bands. Our results imply that a microscopic model capturing strain effects must include a consideration of the orbital nature of bands.

## Abstract (web summary)

The role of orbital degrees of freedom in determining the electronic structure remains obscured. Here, Walkup et al. report strain-induced band structure changes in a topological crystalline insulator SnTe, whose surprising behavior reflects the  orbital nature of bands.

## Introduction

Topological crystalline insulators (TCIs)^1,2^ are a subclass of 3D topological materials, which harbor massless Dirac surface states (SS) tunable by temperature^3,4^ and composition^5^. In contrast to Z_2_ topological insulators^6–8^ where the Dirac point is protected by time-reversal symmetry, the Dirac point in TCIs is protected by a discrete set of crystalline symmetries^1^. This unique coupling between the crystal structure and the Dirac SS provides a route toward controlling the SS dispersion by using different types of structural deformations. For example, lattice distortions that breaks the mirror symmetry protecting the Dirac point enable otherwise massless Dirac SS fermions to acquire mass^9–11^. However, uncovering new pathways for the manipulation of topological SS via structural deformations without breaking the crystalline symmetry protecting the Dirac nodes remains one of the key goals for this system. Strain is one such control knob for TCIs, that is predicted to tune the momentum space position of the Dirac nodes, giving rise to exotic phenomena such as pseudomagnetic fields, unconventional superconductivity^12,13^, and in the extreme case creating a quantum phase transition from the topological to trivial state. Strain control is advantageous both from fundamental and applications perspectives, as it provides a pathway for the in situ manipulation of topological SS via structural deformations without introducing disorder.

The challenges in achieving strain control lie in the difficulty of controllably applying strain, characterizing the type of strain induced, quantifying its magnitude and simultaneously measuring the electronic band structure. In a recent experiment, we used scanning–tunneling microscopy (STM) and spectroscopy to measure the magnitude of local compressive and tensile (biaxial) strain in heteroepitaxial TCI thin films^14^ and were able to correlate the nanoscale variations of biaxial strain to concomitant changes in the momentum space position of the Dirac nodes. Strain in the most general case however, is characterized by a tensor with both diagonal and off-diagonal components, which in different combinations produce biaxial, uniaxial, and shear strain.

Here we use STM to measure all four components of the surface 2D strain tensor with nanometer resolution. We show that in addition to isotropic strain, TCI films also harbor significant uniaxial strain, varying on similar length scales. We correlate the measured strain components with the Dirac-point shifts of the two valleys of surface Dirac fermions, quantitatively characterizing the effects of both biaxial and uniaxial strain on the topological surface states. Measuring uniaxial strain allows us to decouple effects of strain in the *x*- and *y*-directions. By doing this, we reveal an unexpected effect that demonstrates how the orbital nature of an electronic band can have a profound and sometimes counterintuitive effect on its strain response.

## Results

### Spatially varying strain in a heteroepitaxial film

**Fig. 1:**
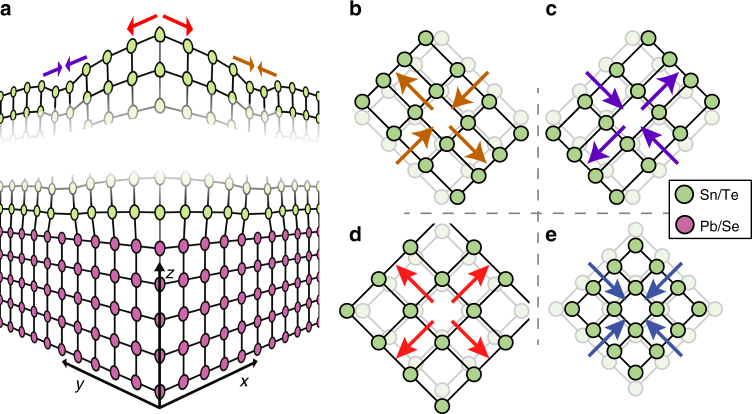
Different types of strain. **a** Schematic of the misfit dislocation network appearing at the interface between the PbSe (001) substrate (purple) and the SnTe film (green). Lattice distortions in the topmost SnTe film in the presence of **b** uniaxial compression along *x*-axis, **c** uniaxial compression along *y*-axis, **d** biaxial tensile strain and **e** biaxial compressive strain. The color of arrows in **b**–**e** indicates the position on the sample in **a**

**Fig. 2:**
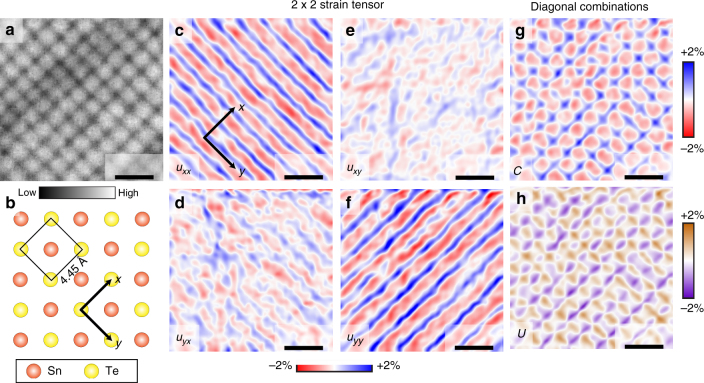
Spatial distribution of different types of strain. **a** STM topograph of ~130 nm square region of the sample (*V*_set_ = −50 mV, *I*_set_ = 200 pA), showing the strain-generated topographic superlattice with period of ~ 15 nm. **b** Schematic of the (001) surface of SnTe. Arrows in **b** denote the *x*- and *y*-axes, which have the same orientation in all panels, as shown again in **c**. **c-f** The components of the 2 × 2 strain tensor ∇**u**(**r**) extracted from the topograph in **a**. *u*_*ij*_ denotes (∂*u*_*i*_)/(∂*r*_*j*_). **g** The biaxial strain map *C*. **h** The uniaxial strain map *U*. All scale bars are 30 nm

To generate strain, we grew (001)-oriented thin films of the TCI SnTe on non-topological insulator PbSe (001) substrates. Such heterostructures exhibit grid-like quasi-periodic arrays which arise from misfit dislocations running in two perpendicular directions, which are associated with strain patterns near the interface (Fig. 1)^14–16^ These sub-surface dislocations manifest themselves as linear dips or troughs in the STM topographs directly above the line of the dislocation^15,17^ (Fig. 1a). As shown in our previous work^14^, the ~3% lattice mismatch between the film and the substrate results in a spatially inhomogeneous strain field. This could lead to areas of compressive, tensile, or uniaxial strain as depicted in Fig. 1b–e. To quantify this strain, we start with an atomically resolved STM topograph *T*(**r**) (Fig. 2a) and subject it to a geometrical phase analysis method^18^, closely related to the Lawler-Fujita drift correction procedure^19^ (see Supplementary Note I). This method calculates the displacement field **u**(**r**), which defines the distance by which the imaged lattice is shifted from perfect periodicity, with a (parametric) spatial resolution of 3–4 lattice constants. After background subtraction the derivatives of **u** along the lattice vectors (Fig. 2b) directly give the strain tensor (Supplementary Note I).

The spatially resolved components of the strain tensor—*u*_*ij*_(**r**) ≡ ∂*u*_*i*_(**r**)/∂*r*_*j*_—are shown in Fig. 2c–f. A careful comparison of these results with the topographic image (Fig. 2a) reveals the overall strain distribution depicted in Fig. 1. Let us first consider the diagonal components of the strain tensor (Fig. 1c, f). By comparing with the cartoon in Fig. 1a, we see that the troughs (crests) running along a particular direction are associated with a uniaxial compression (tension) in the perpendicular direction (Fig. 2c, f). Combining these elements of the stain tensor, we can see how the same sample can host both biaxial as well as uniaxial strain. At the intersection of two troughs (crests) running in perpendicular directions, the strain amounts to an isotropic squeeze (stretch), respectively (Fig. 1d, e). In a region where a trough in one direction coincides with a crest in the other, the strain is highly anisotropic: the lattice is squeezed perpendicular to the trough but stretched parallel to it (Fig. 1b, c). To show these features of the strain distribution more explicitly, we introduce the spatially resolved (isotropic) biaxial compression *C*(**r**) ≡ (*u*_*xx*_ + *u*_*yy*_)/2 (Fig. 2g), and the antisymmetric uniaxial strain *U*(**r**) ≡ (*u*_*xx*_ − *u*_*yy*_)/2 (Fig. 2h) both of which show periodic modulations which visually correlate with the topographic modulations shown in Fig. 2a.

### Quasiparticle interference as a measure of band structure

**Fig. 3:**
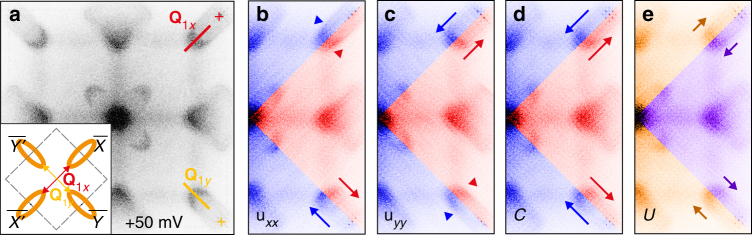
Strain-filtered Fourier transforms of d*I*/d*V*. **a** The Fourier transform of d*I*/d*V* acquired in the 130 nm area shown in Fig. 2a; the indicated features **Q**_1*x*_ and **Q**_1*y*_ represent scattering across the center of the Brillouin zone between the inner portions of the pockets (inset) at $\bar X$, $\overline X \prime$, and $\bar Y$, $\overline Y \prime$ respectively. **b**–**e** The Fourier transforms of masked d*I*/d*V*, with masks chosen to capture the maxima (blue in **b**–**d**, orange in **e**, and minima (red in **b**–**d** and purple in **e**) of *u*_*xx*_, *u*_*yy*_, *C*, and *U*, respectively; the arrows are guides to the eye. In **b**–**e**, the blue (orange) and red (purple) subsets correspond to masks coincident with the blue (orange) and red (purple) areas of Fig. 2c, f, g, h, respectively. For details of the masking procedure, see Supplementary Note II

To study the effect of strain on the electronic structure, we use quasiparticle interference (QPI) mapping, which can be used to measure the band structure of quasi 2D bands or 2D surface states^20^. QPI imaging relies on the elastic scattering of quasiparticles on the surface of a material, which produce standing waves in the density of states. These standing waves appear as oscillations or ripples in the measured local density of states, *G*(**r**,*V*) ≡ d*I*/d*V*(**r**,*V*), with wavevector **q** = **k**_**i**_−**k**_**f**_, where **k**_**i**,**f**_ are the initial and final momenta of the scattered quasiparticles. These **q**-vectors can be directly extracted from the Fourier transform of *G*(**r**,*V*), and reveal the momentum space position of the underlying bands. The surface states of SnTe(001) consist of a pair of Dirac cones near each $\bar X$ and $\bar Y$ point. Each pair undergoes a Lifshitz transition so that at the energies shown here (~200–250 meV below the Dirac point), the constant-energy-contours resemble the ellipses around each $\bar X$ and $\bar Y$ point (inset of Fig. 3a)^2,21.^ In this work, we focus on the vectors labeled **Q**_1*x*_ and **Q**_1*y*_ in Fig. 3a, which represent scattering across the $\it\bar \Gamma$ point between two ellipse-like bands on the opposite sides. Because **Q**_1*x*_ and **Q**_1*y*_ each pertain to a single valley of Dirac fermions, we can valley-decouple the effect of any given type of strain. The effect of the isotropic strain *C* on the Dirac surface states was previously investigated by us^14^; here we complete the picture by considering the effects of uniaxial strain. The off-diagonal elements of the strain tensor (Fig. 2d,e) are relatively weak and lack a clear spatial pattern; in what follows they are neglected.

### Spatially filtered QPI

**Fig. 4:**
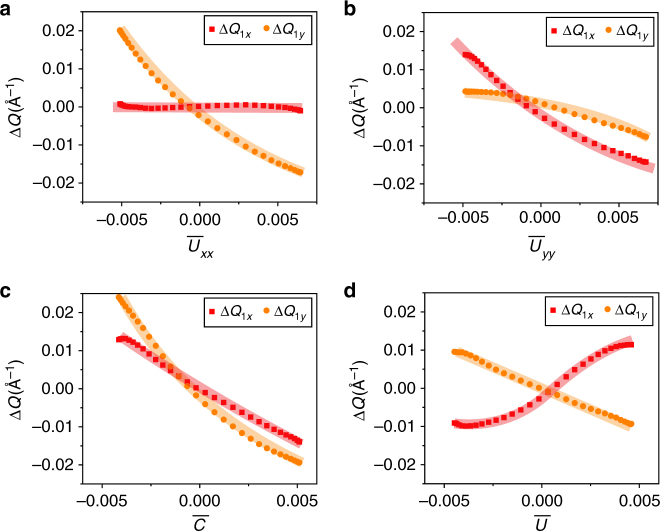
The shift of the QPI peaks as a function of average strain. The absolute change in the QPI scattering wavevectors Δ*Q*_1*x*_ and Δ*Q*_1*y*_ plotted against the average values of: **a** *u*_*xx*_, **b** *u*_*yy*_, **c** *C*, and **d** *U*

The influence of the strain on the surface states was determined by spatially filtering *G*(**r**,*V*) using a set of masking functions, and extracting the **q**-vectors from the Fourier transforms of the masked data. We employ masks designed to isolate the diagonal components *u*_*xx*_(**r**) and *u*_*yy*_(**r**) (Fig. 2c, f), as well as the linear combinations *C*(**r**) and *U*(**r**) (Fig. 2g, h). For the former, the mask consists of a series of linear strips, one for each topographic trough, along the *y*- and *x*-axes respectively; for the latter the masks are two-dimensional, quasi-circular blobs, one for each unit cell of the dislocation superlattice; these masks are shown schematically in Supplementary Figure 1. We note that using two different types of mask provides redundant information that was used to check the validity of our approach. Each masking filter is normalized to contain 33% of the total d*I*/d*V* signal, but is parameterized so that it can be swept smoothly through space (Supplementary Note II), allowing us to obtain smooth curves of **Q**_1*x*,*y*_ versus the mask-averaged strain (Fig. 4).

## Discussion

To demonstrate the phenomenology qualitatively, we first compare FTs of the masked d*I*/d*V* $G_m^i({\mathbf{r}})$ for two non-overlapping masks capturing the extreme values of each type of strain (Fig. 3b–e). Consistent with our previous work^14^ as well as theoretical predictions^12^ we find that compressive biaxial strain causes both **Q**_1*x*_ and **Q**_1*y*_ to shift toward the center of the FT by approximately the same amount (Fig. 3d). This reflects the stretching of the ellipse-like pairs of bands at $\bar X$ and $\bar Y$, respectively, which is caused by the momentum space shift of the Dirac cones toward $\it {\bar \Gamma }$. From Fig. 3e we can now also see the effects of the uniaxial strain and find that, as predicted by theory, uniaxial strain shifts **Q**_1*x*_ and **Q**_1*y*_ in the opposite directions.

Our data allow us to obtain quantitative information on the influence of the strain on the position of the Dirac nodes. Theory predicts^12^ that in the (001) surface states of TCIs, strain acts as a gauge field which shifts the Dirac points linearly. Leaving aside the shear terms, one obtains the expression

1 $$\begin{array}{l}A_x = \left( {\alpha _1u_{xx} + \alpha _2u_{yy}} \right)\\ A_y = \left( {\alpha _1u_{yy} + \alpha _2u_{xx}} \right),\end{array}$$

where *A*_*x*,*y*_ is the inward shift of the Dirac point near $\bar X$, $\bar Y$, respectively. However, the magnitude and the sign of the coefficients *α*_1,2_ are yet to be determined experimentally. To calculate these constants, we sweep the two masking functions through the d*I*/d*V* maps and the topography. At each mask position, the mask-averaged value of each component of the strain tensor^14,22^ is calculated and the corresponding Fourier transform of the masked area is obtained (Supplementary Note III and IV). Figure 4 shows the changes in the **Q**-vectors Δ**Q**_1*x*_ and Δ**Q**_1*y*_ as a function of the strain tensor components *u*_*xx*_ and *u*_*yy*_ as well as the linear combinations *C* and *U*. From linear fits to the plots in Fig. 4, we obtain *α*_1_=0.3 Å^−1^, and *α*_2_=1.5 Å^−1^; the error is on the order of 0.1 Å^−1^, comparable to the discrepancy between the results obtained by fitting Fig. 4a, b, and those obtained from Fig. 4c, d (Supplementary Table 1).

In studying the spatially dependent strain tensor components, we find an intriguing feature. As shown in Fig. 3b, c, strain applied along *x* (Fig. 3b) causes the most prominent change in the orthogonal direction, along *y*, by shifting the position of the **Q**_1y_. Conversely, the strain applied along *y* causes the most pronounced change in the orthogonal direction, by shifting the position of **Q**_1*x*_ (Fig. 3c); this is also reflected in the relative sign of the shifts under uniaxial strain (Fig. 3e; Fig. 4d). This leads us to the question: what is the origin of this seemingly counterintuitive response of the Dirac cones?

**Fig. 5:**
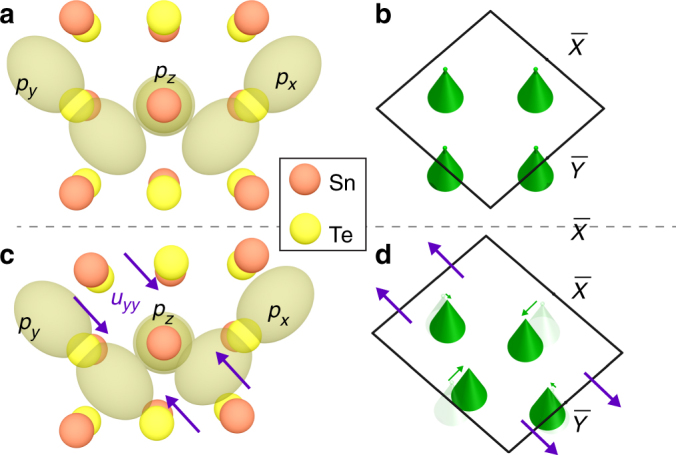
The effects of orbital overlap on the electronic band structure in TCIs. Schematic of the relevant orbitals: **a** in the absence of any distortion, and **c** under strain along *y*-axis. The view shown is a top view of the (001) direction of the lattice with the top two layers represented by slightly shifted atoms. The *P*_*x*_ and *P*_*y*_ orbitals belong to the top layer Te atoms, whereas the *p*_*z*_ orbital belongs to a second layer Te atom. **b**,** d** The positions of the four Dirac cones (green) within the first Brillouin zone related to the atomic structure in **a**, **c**, respectively. As seen in **c**, strain in *y*-direction increases the “overlap” of *p*_*x*_ and *p*_*z*_ orbitals, whereas the overlap of *p*_*y*_ and *p*_*z*_ does not significantly change

To answer this question, we carried out Tight-binding (TB) model calculations as described below (see Supplementary Note V for details). In general, the momentum space position of the Dirac nodes around each $\bar X$ point in TCIs is directly related to the bulk band gap at $\bar X$ – the larger the bulk band gap, the further the Dirac node is from $\bar X$. Therefore, to determine what drives the evolution of Dirac nodes, we need to consider the hopping terms that influence the magnitude of the bulk gap. By changing the magnitude of each hopping term and recording its effect, we determined the hopping terms that have the largest effect on the bulk band gap. We find that the hopping terms *t*_1_ and *t*_2_, which quantify the hopping between *p* orbitals on Te atoms on two particular sites as shown in Fig. 5, have the most impact on the band structure (Supplementary Note V). To first approximation, in order to model strain effects on the band gap, it is sufficient to determine changes in *t*_1_ and *t*_2_ due to strain.

To describe SnTe under strain in one axis, we first build a simple TB model under Slater and Koster’s method. For simplicity, we only consider hoppings between *p* orbitals the nearest Sn–Te, Sn–Sn, Te–Te, which are labeled as *V*_Sn − Te_, *V*_Sn–Sn_ and *V*_Te–Te_. For each hopping, we could decompose it into *σ* and *π* bounds:

2 $$\begin{array}{*{20}{l}} {V_{\rm AB}^\sigma } \hfill & = \hfill & {\alpha _{\rm AB}^\sigma \ast {\rm e}^{ - \beta r_{\rm AB}} \ast \sqrt {rd_{\rm A} \ast rd_{\rm B}} } \hfill \\ {V_{\rm AB}^\pi } \hfill & = \hfill & {\alpha _{\rm AB}^\pi \ast {\rm e}^{ - \beta r_{\rm AB}} \ast \sqrt {rd_{\rm A} \ast rd_{\rm B}}, } \hfill \end{array}$$

where *r*_AB_ is distance between A and B atoms, $\alpha _{\rm AB}^\sigma$ and $\alpha _{\rm AB}^\pi$ decide the strength of related hoppings, *β* decides the decay rate of this hopping with distance, *rd*_A_ and *rd*_B_ are constants related to each atom. The onsite energy of each atom is labeled as *m*, and the effects of spin-orbit coupling are described by *λ*. Using the parameters listed in the Supplementary Table 2, the above TB model roughly reproduces the results from DFT, as shown in Supplementary Figure 6c.

To understand our experimental results, let us consider a two-center approximation in the tight-binding formalism in which the hopping terms *t*_1_, *t*_2_ can be written as:

3 $$\begin{array}{l}t_1 = \frac{{xz}}{r}\left( {pp\sigma - pp\pi } \right)\\ t_2 = \frac{{yz}}{r}\left( {pp\sigma - pp\pi } \right),\end{array}$$

where *ppσ* and *ppπ* are the projected *σ*-bond and *π*-bond between *p* orbitals, *r* is the distance between two Te atoms while *x*, *y*, and *z* are the distance projected on the *x*, *y*, and *z* axis, respectively. Note that changing *t*_1_ and *t*_2_ primarily shifts the energy level of the conduction bands. This is because the conduction bands arise from the Te orbitals, whereas the valance bands come from Sn orbitals, which are not affected. To understand our observations, it is also important to realize that the conduction bands at $\bar X$ are mainly composed of *p*_*x*_ orbitals, whereas those at $\bar Y$ mainly consist of *p*_*y*_ orbitals (Fig. 5). When *y*-axis is squeezed, one can show that the change of *t*_2_ is greater than that of *t*_1_ by replacing *y* by *y* − *dy* and calculating the leading term of *dx* (also see Supplementary Note V). Qualitatively, this can be seen by looking at the overlap of orbitals as the lattice is strained (Fig. 5c). The overlap between *p*_*y*_ and *p*_*z*_ orbitals does not significantly change, but the overlap between *p*_*x*_ and *p*_*z*_ is clearly larger. Thus, strain along *y*-axis does not significantly affect the bulk band gap at $\bar Y$, but causes a change in the bulk band gap at $\bar X$. This implies that the Dirac node shift of the Dirac cones near $\bar X$ will be significantly stronger than the shift of the Dirac cones near $\bar Y$ (Fig. 5d), which is exactly confirmed in our experiments.

By correlating measurements of the 2D nanoscale strain tensor with changes in the band structure in momentum space, we have discovered that TCIs display a highly non-trivial strain response. Our tight-binding calculations reveal that our experimental observations can be explained by strain-induced changes in hopping parameters that are strongly influenced by the directional nature of *p*orbitals in topological materials, which make the hopping necessarily anisotropic. We find that this anisotropy can have a profound and counterintuitive effect on the strain response of the band structure in momentum space. These results imply that any microscopic model for strain effect must include a consideration of the orbital nature of the bands.

## Methods

### Thin film synthesis

The thin films used in our experiment were synthesized using e-beam evaporation in an ultra-high vacuum chamber directly attached to the STM. SnTe thin films were deposited at 300 °C at the growth rate of ~ 2 monolayers per minute for a total ~40 ML thickness, as determined by the thickness of SnTe thin films grown on Si(001) under the same conditions. The PbSe single crystal used as a substrate was grown by the self-selecting vapor growth method and cleaved at room-temperature in ultra-high vacuum along the (001) direction to expose a pristine surface free of contaminants.

### STM details

Within minutes after the completion of the growth process, the films were inserted into the STM head where they were held at 4 K for the duration of the experiments presented in this work. All STM data were acquired at ~ 4.5 Kelvin using a Unisoku USM1300 STM. d*I*/d*V* maps were acquired using a standard lock-in technique with 10 meV excitation amplitude at 1488 Hz.

### Data availability

All relevant data are available on request from the authors.

## Electronic supplementary material

Supplementary Information

Description of Additional Supplementary Files

Supplementary Movie 1

Supplementary Movie 2
