# Supplementary material for: Interplay of orbital effects and nanoscale strain in topological crystalline insulators
Source: Nat Commun. 2018 Apr 19;9:1550. doi: 10.1038/s41467-018-03887-5 (PMC5908802; doi:10.1038/s41467-018-03887-5)
Supplement: Supplementary file 1 — Supplementary Information [file 41467_2018_3887_MOESM1_ESM.pdf]

Supplementary Figures

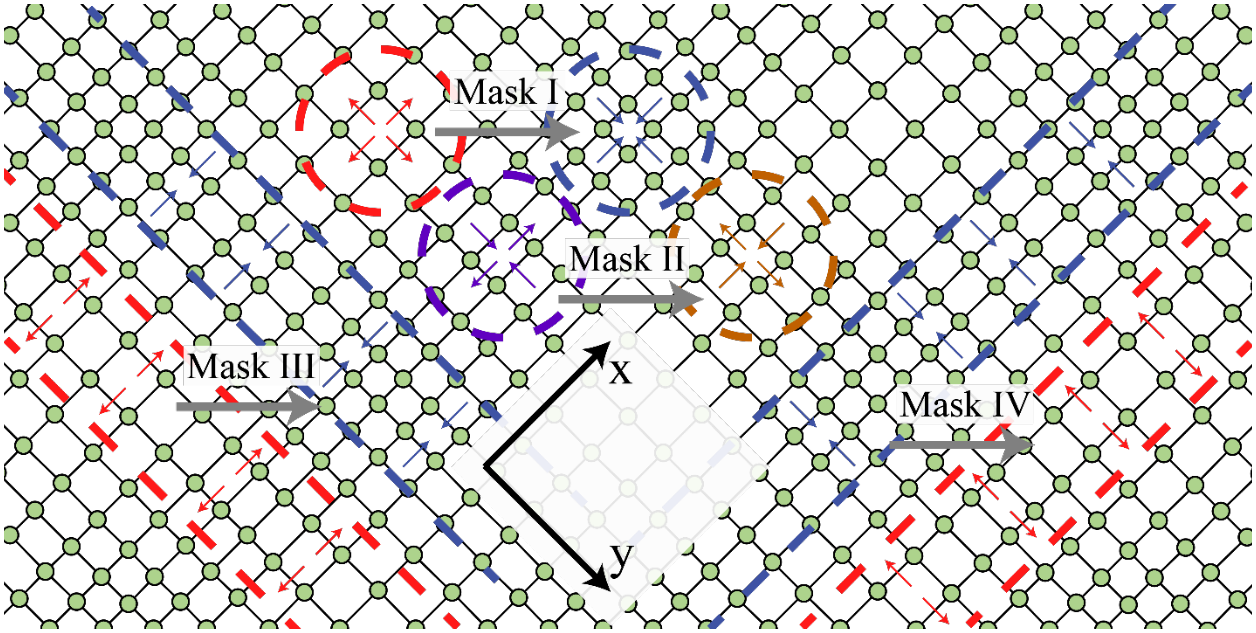

Supplementary Figure 1. Schematic of the masking procedure. *A cartoon of the strain superlattice observed on the SnTe surface (green circles). Superimposed are the four types of spatial filters used to extract the Fourier transforms of Fig. 3(b)-(e), and the ΔQ vs. average strain plots of Fig. 4. Mask I filters the isotropic strain C, concentrated at the intersections of topographic troughs (blue) or topographic peaks (red). Mask II filters the purely uniaxial strain, which is greatest where troughs in one direction intersect peaks in the other (purple and orange). Mask III and IV are linear, running parallel to the troughs in the x- and y-directions, and filtering the strain components u_{xx} and u_{yy} respectively. Masks I,II,III,IV relate to panels (d),(e),(b),(c) respectively of Fig. 3; the color scale of the FFT matches that of the mask. The masks can be translated continuous through space (grey arrows), producing the curves shown in Fig. 4.*

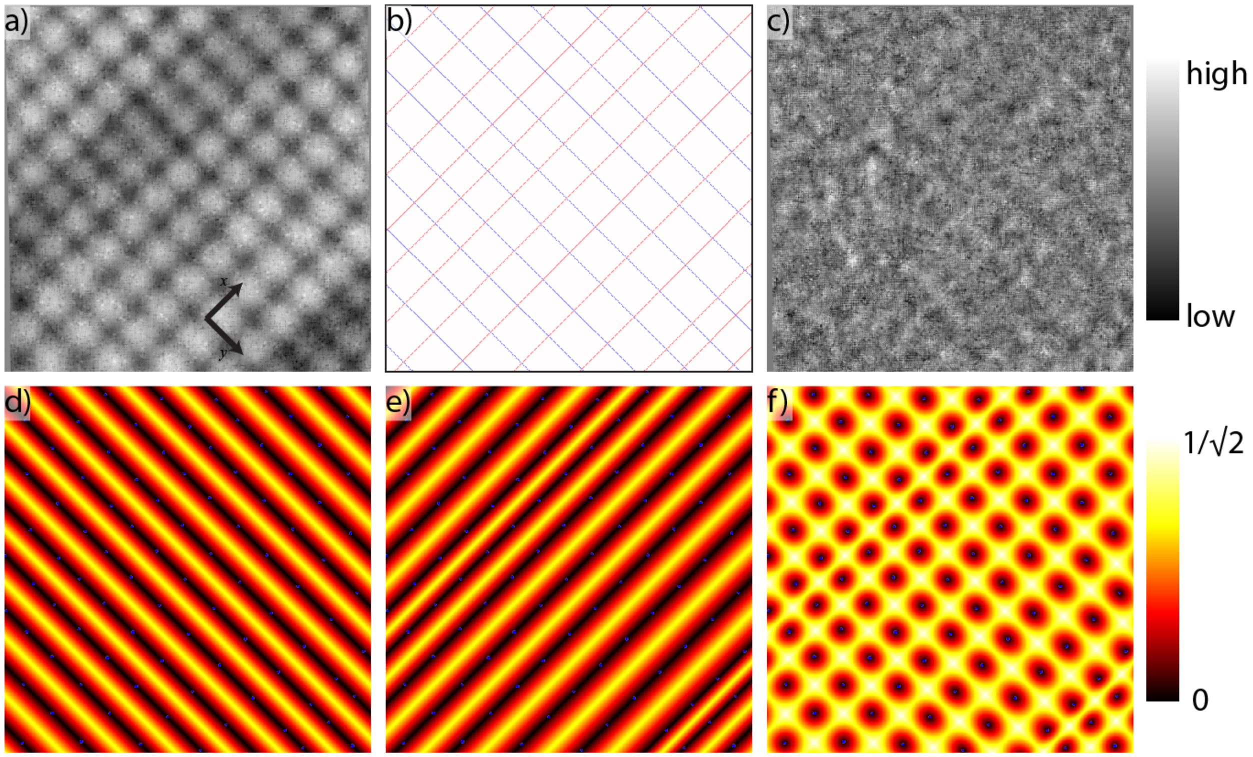

Supplementary Figure 1. Masking function ingredients. *(a) The drift-corrected topography at +50 mV. (b) The grid lines extracted from the topography, defining the dislocation coordinate system. (c) The raw dI/dV, simultaneously acquired with the topography in (a). (d)-(f) are distances defined in the main text, for $\mu, \nu = 0$. The blue dots are the integer points of the coordinate system (the line-crossings in (b)), and approximately coincide with the intersection of troughs in (a). (d), (e), (f), are d_a, d_b, $d(\mathbf{r})$ respectively.*

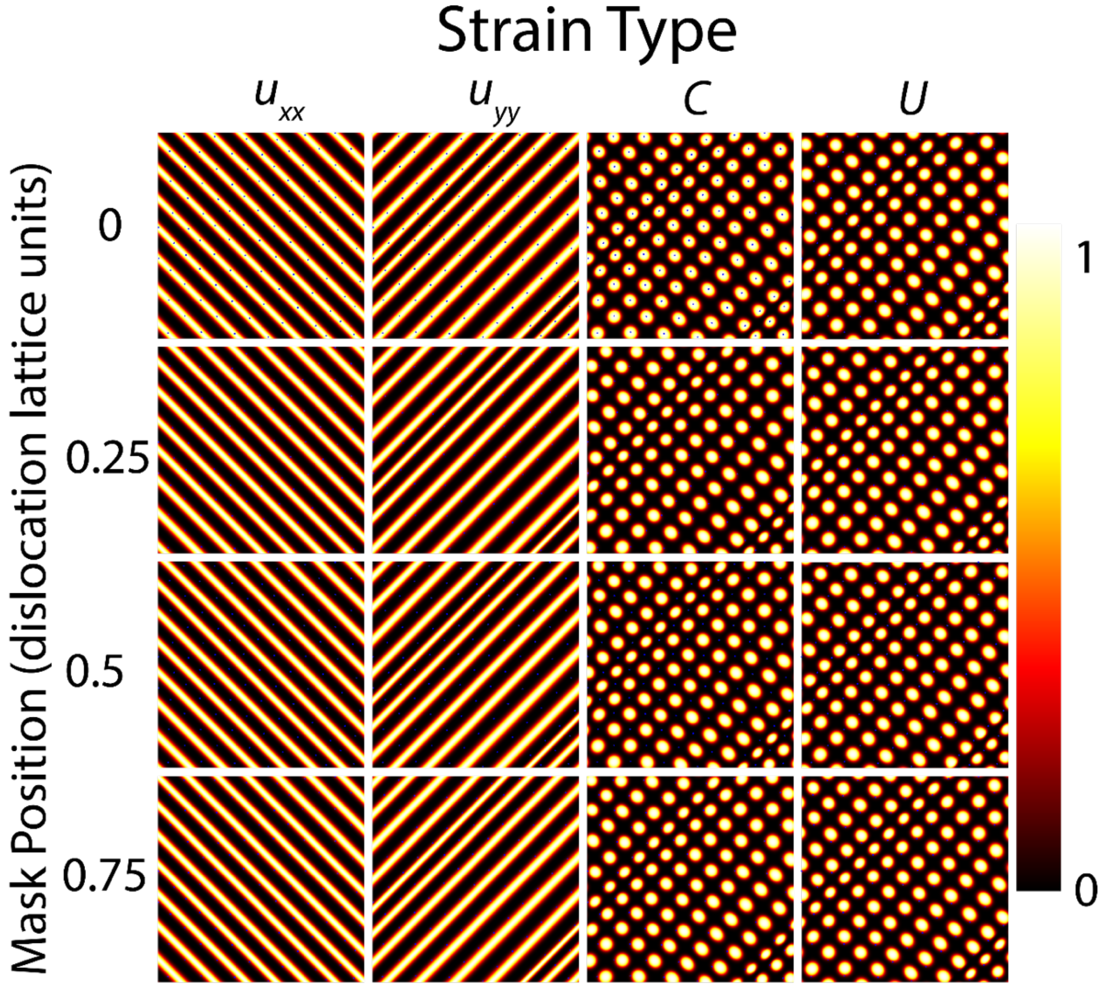

Supplementary Figure 2*. *Masking functions. *The masking functions used are shown for each type of strain, and for important values of the mask position* η*. The trough junctions (grid points of the dislocation lattice) are blue dots as in* ***Supp. Fig. 2****. For each strain type (using the sign conventions in this paper) the largest average strain occurred at* $\eta \approx 0$*, and the smallest (negative) at* $\eta \approx 0.5$*;* $\eta = 0.25$ *and* 0.75 *were approximately neutral. All masks move to the left with increasing* η*.*

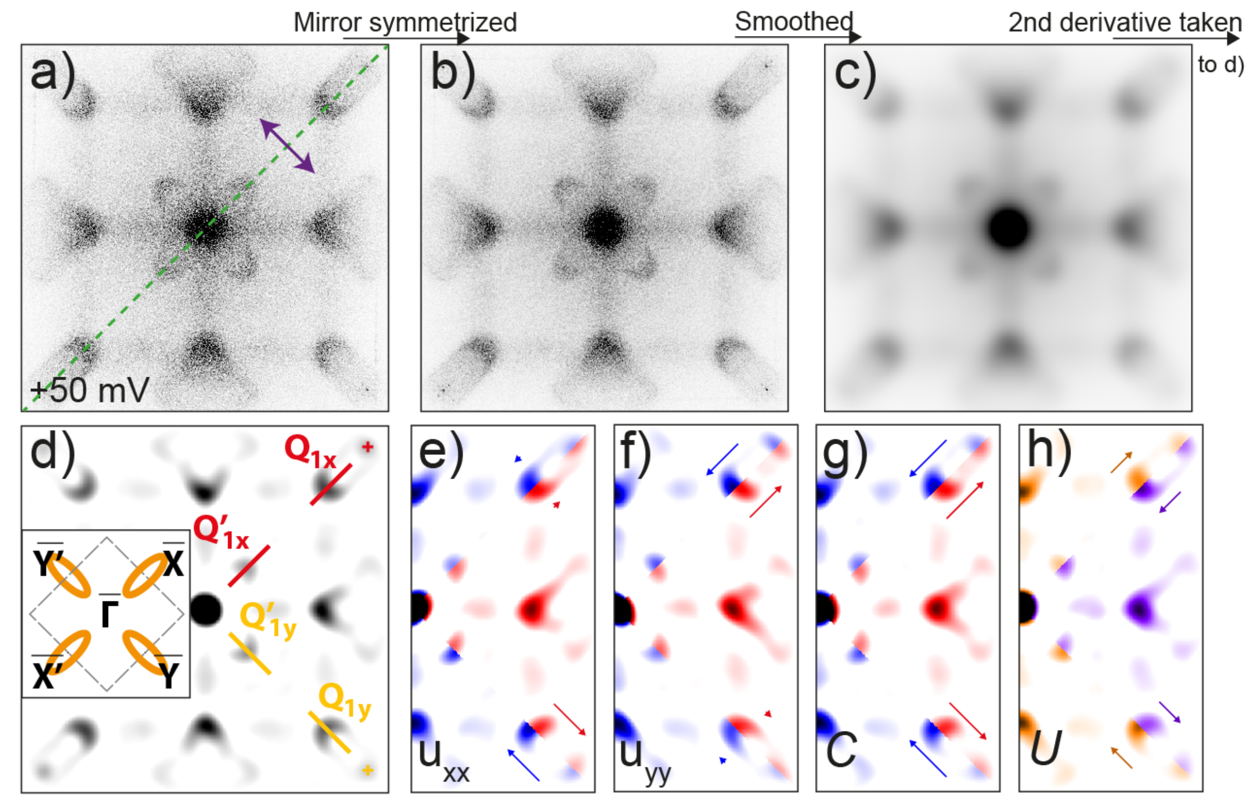

Supplementary Figure 3. Pre-fit processing of the Fourier transforms. *The raw FFT (a) is symmetrized (b) by reflection about the* x*-axis (green dashed line in (a)). We smooth sufficiently to wash out the ringing introduced by the masking functions (c), and take the second derivative (d). The result clearly reveals the intra-pocket scattering feature, denoted* $\boldsymbol{Q}_1'$*. Panels (e)-(f) are thus-processed FFTs of the masked dI/dV, for mask position* $\eta = 0$ *(blue and orange) and* $\eta = 0.5$ *(red and purple); as in* ***Fig. 3 (b)-(e)*** *of the main text.*

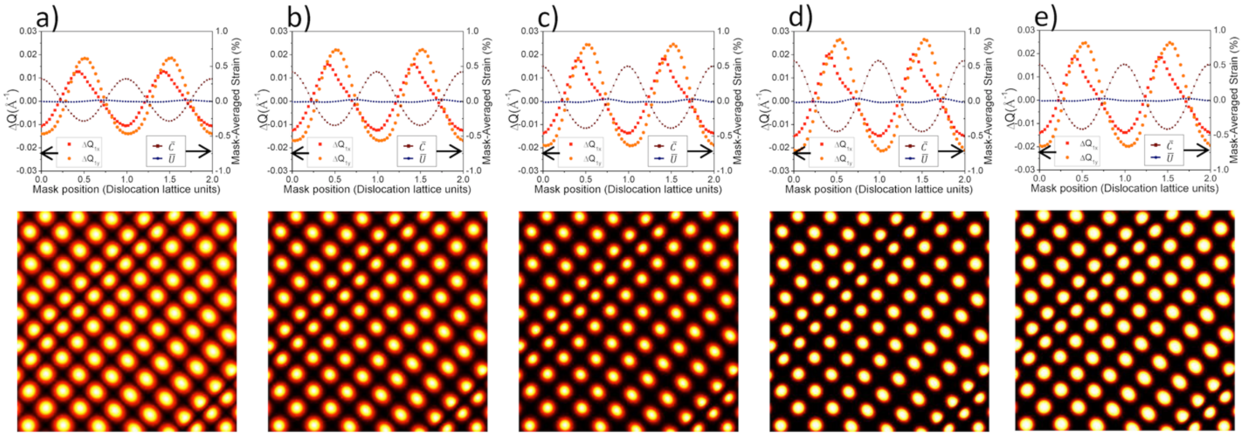

Supplementary Figure 4. Masking for compressive strain with different masks. *The five panels show the variation in* $\Delta Q_{1x,y}$, $\bar{C}$ *and* $\bar{U}$ *against* η *for the masks, whose values for* $\eta = 0$ *are shown below the corresponding graph (color scale same as* ***Supp. Fig. 2****). (a),(b),(c) are Gaussian masks designed to capture 1/3rd, 1/4th, 1/5th of the total spectral weight respectively. (That is,* $M(\boldsymbol{r}) = e^{-d^2/\lambda^2}$ *for* $\lambda = 0.338, 0.286, 0.253$ *respectively.) (d) and (e) are masks employing the Fermi function with T=0.05 capturing 1/5th and 1/4th respectively of the total spectra weight. (*$\varphi = 0.267$ *and 0.235 respectively.) The mask shown in (e) is the same as that used in the main paper.*

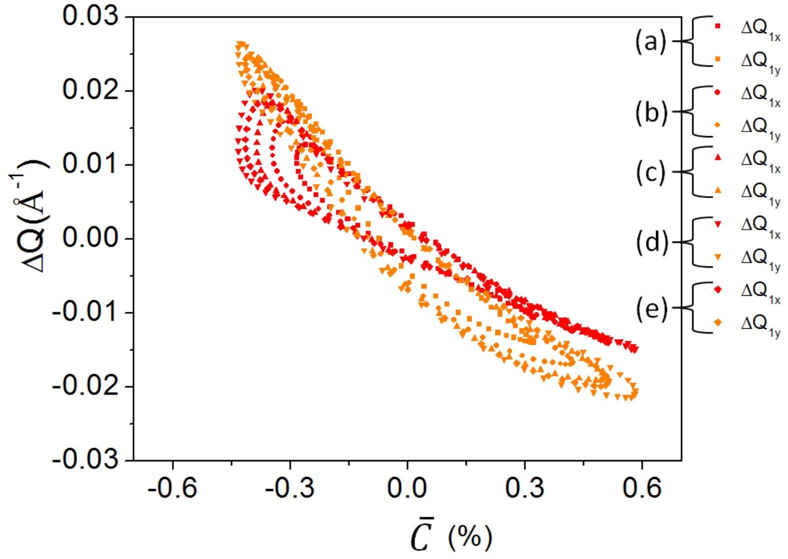

Supplementary Figure 5. ΔQ_1 vs compressive strain for different mask tyes. *The average compressive strain and ΔQ_1 values are plotted for the five mask types shown in **Supp. Fig. 4**; the shapes are indicated in the legend and the letter labels indicate the corresponding panel of **Supp. Fig. 4**. The slopes of the linear fits to the data are for **ΔQ_{1x}** -3.53, -3.28, -3.11, -2.93, -3.04 respectively; the slopes for **ΔQ_{1y}** are -5.33, -5.02, -4.76, -4.50, -4.70 respectively. All slopes are in $Å^{-1}$.*

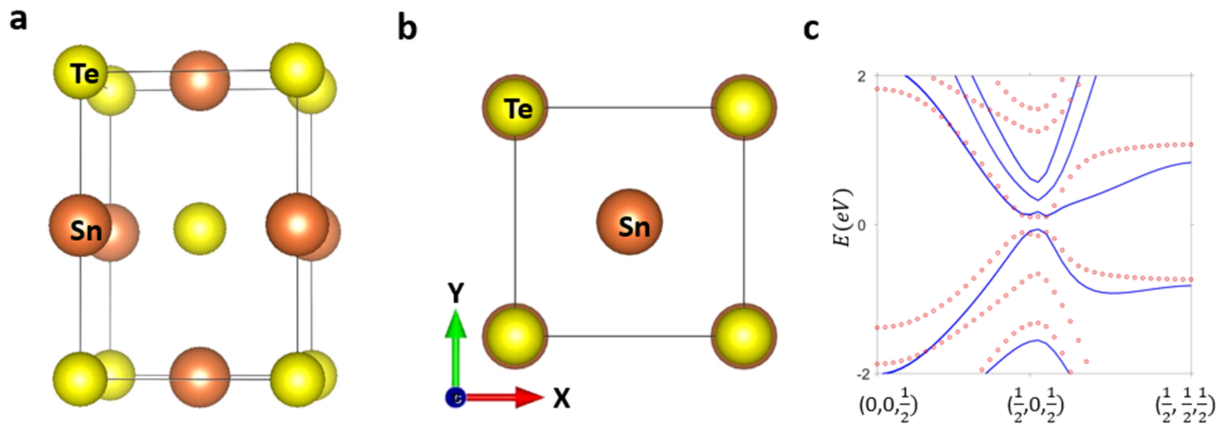

Supplementary Figure 6. A simple TB model *(a) The crystal structure used in building the TB model (b) Top view of the crystal (c) The blue bands are from DFT and the red circles are calculated from the TB model with the above parameters.*

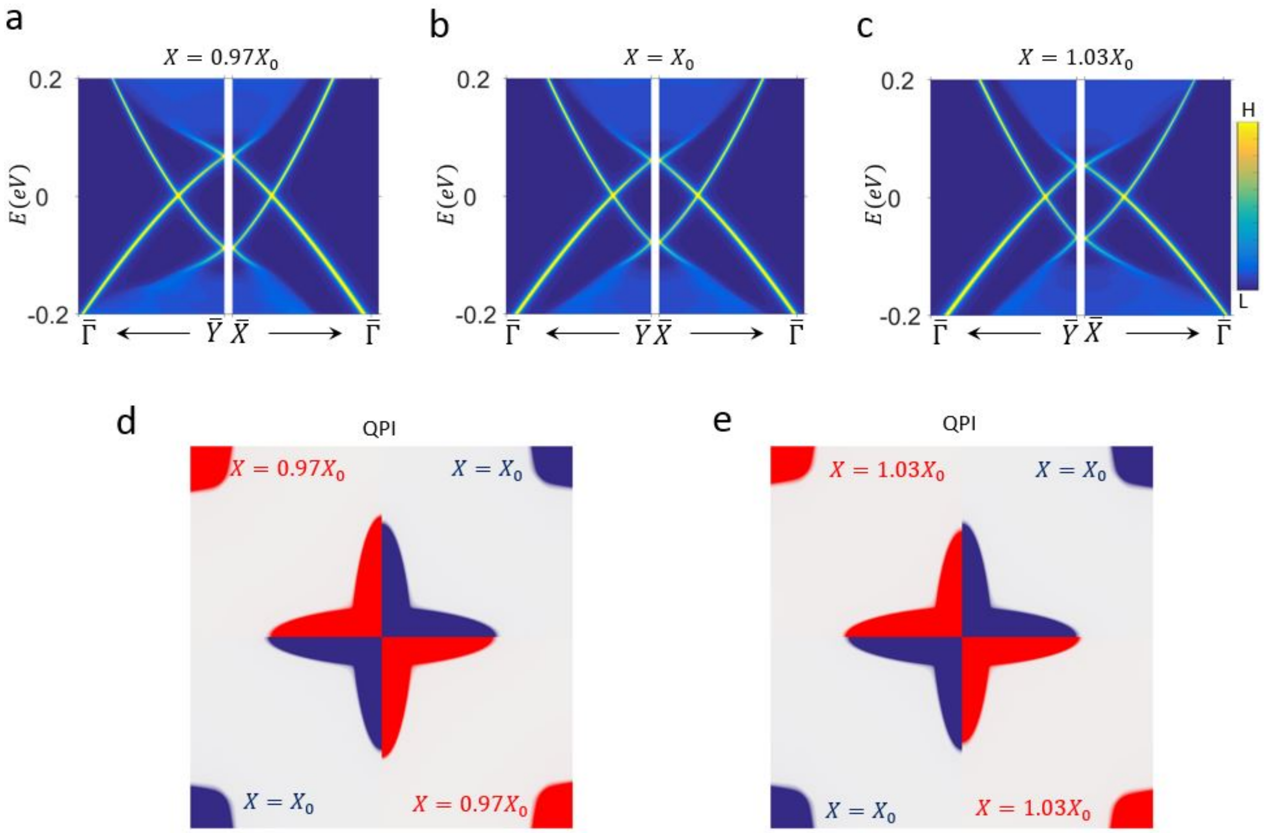

Supplementary Figure 7. Calculated surface states and QPI patterns of SnTe under different strain along x-axis. *(a, b, c) The E-k dispersions along y and directions when the x-axis is 97%, 100%, 103% of the original one, respectively. (d) The comparison between the two cases where the x-axis is squeezed by 3% (colored by red) and the x-axis remains the same (colored by blue). The QPI patterns along y direction are obviously increased when the length of x-axis is decreased. (e) The comparison between the two situations where the x-axis is stretched by 3% (colored by red) and the x-axis remains the same (colored by blue).*

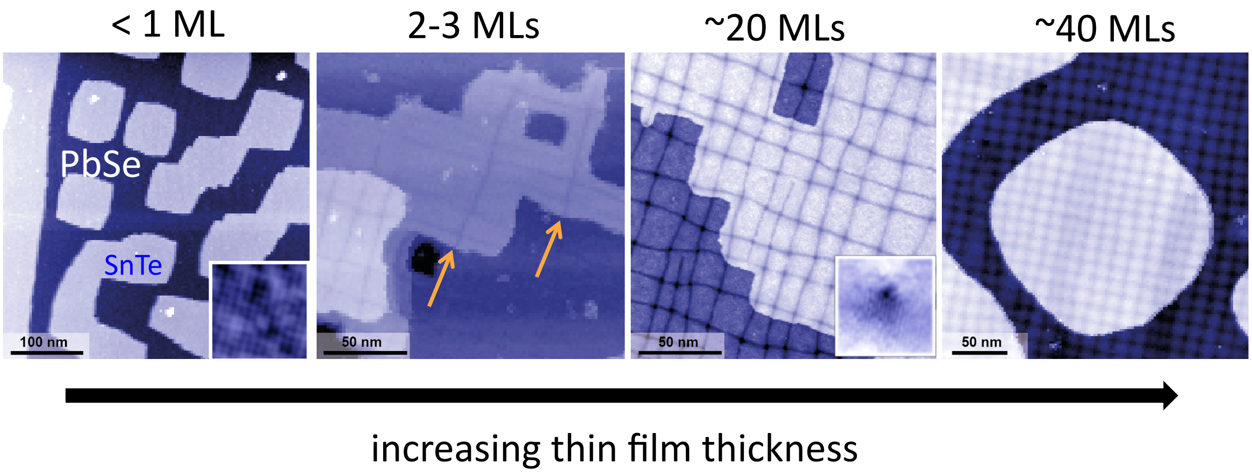

Supplementary Figure 8. Evolution of SnTe thin films with thickness. *We show four STM topographs of SnTe films with increasing thickness. Initially, SnTe forms as pseudomorphic islands (left). As thickness increases, edge dislocations nucleate at the interface, appearing as dark lines in the topography beginning at 2-3ML (arrows), and continuing to ~40ML. For details, see Ref. 3.*

Supplementary Tables

Slopes (Å^{-1})			Coefficients (Å^{-1})			
	ΔQ_{1x}	ΔQ_{1y}		From u_{xx} & u_{yy}	From C & U	Average
vs. u_{xx}	-0.05	-3.15	α_1	0.28	0.37	0.3
vs. u_{yy}	-2.53	-1.04	α_2	1.42	1.57	1.5
vs. C	-3.04	-4.7	β	1.70	1.94	1.8
vs. U	2.63	-2.14	γ	-1.14	-1.20	-1.2

Supplementary Table 1. *Fitting slopes and extracted coefficients for Eq. (1) and (2).*

α^{σ}_{Sn-Te}	α^{π}_{Sn-Te}	α^{σ}_{Sn-Sn}	α^{π}_{Sn-Sn}	α^{σ}_{Te-Te}	α^{π}_{Te-Te}
6	-3	-1.2	0.3	1.2	-0.3

m_{Sn}	m_{Te}	β	rd_{Sn}	rd_{Te}	λ_{Sn}	λ_{Te}
1.507	0	0.51	1.6	1	0.35	0.55

Supplementary Table 2. *Tight-binding parameters used.*

Supplementary Note I

The image-analysis method we used to extract the strain is derived in Ref. [1] and is closely related to the Lawler-Fujita distortion correction algorithm.[2] We describe it briefly below.

The atomic lattice in an STM topography can be expanded in a Fourier series. To first-order, $T(\mathbf{r}) \approx A_1 e^{i\mathbf{k}_1\cdot\mathbf{r}} + A_2 e^{i\mathbf{k}_2\cdot\mathbf{r}} + \text{c.c.}$ where $\mathbf{k}_1$and $\mathbf{k}_2$ are the basic reciprocal lattice vectors. When the lattice is distorted the maxima corresponding to the atoms shift from $\mathbf{R}$ to $\mathbf{R}+\mathbf{u}(\mathbf{R})$, so that the Fourier expansion becomes

$$T(\mathbf{r}) \approx A_1 e^{i\mathbf{k}_1\cdot(\mathbf{r}+\mathbf{u})} + A_2 e^{i\mathbf{k}_2\cdot(\mathbf{r}+\mathbf{u})} + \text{c.c.}$$

When we multiply by $e^{-i\mathbf{k}_1\cdot\mathbf{r}}$, we obtain

$$T_1(\mathbf{r}) \approx A_1 e^{i\mathbf{k}_1\cdot\mathbf{u}} + A_2 e^{i\mathbf{k}_2\cdot(\mathbf{r}+\mathbf{u})-\mathbf{k}_1\cdot\mathbf{r}} + \cdots$$

All the terms in T_1 besides the first oscillate rapidly; they vanish upon smoothing with some length scale L, leaving $\tilde{T}_1(\mathbf{r};L) \approx A_1 e^{i\mathbf{k}_1\cdot\mathbf{u}}$; we similarly obtain $\tilde{T}_2(\mathbf{r};\mathrm{L}) \approx A_2 e^{i\mathbf{k}_2\cdot\mathbf{u}}$. Generally, extracting $\tilde{T}_1$ and $\tilde{T}_2$ requires that in the vicinity of the Bragg peak the only significant spectral weight be due to the atomic lattice; this vicinity is of order $1/L$.

The strain is given by the derivatives of $\mathbf{u}$. To find these, we first normalize $\tilde{T}_1$ and $\tilde{T}_2$ throughout all space ($\tilde{N}_1 \equiv \tilde{T}_1/|\tilde{T}_1| = e^{i\mathbf{k}_1\cdot\mathbf{u}}$), then take the gradient and multiply by the complex conjugate:

$$\nabla(\mathbf{k}_1 \cdot \mathbf{u}) = \frac{1}{i}\tilde{N}_1^{\star}\,\nabla\tilde{N}_1$$

and similarly for $\nabla(\mathbf{k}_2 \cdot \mathbf{u})$. (cf. [1] Appendix D.) To go from the derivatives $\nabla(\mathbf{k}_{1,2} \cdot \mathbf{u})$ to the $\nabla\mathbf{u}$ used in this paper (Fig. 2 (c-f)) requires normalization and some additional linear algebra, which is trivial if $\mathbf{k}_{1,2}$ already form a square lattice.

In the STM literature the main contributions to $\mathbf{u}$ have been not strain, but technical factors specific to STM: piezoelectric effects (nonlinearities, hysteresis, curvature of the tube) and thermal drift.[2] The hysteretic effects are often especially pronounced at the beginning of an STM scan; for the data in this paper we cropped the first 24 lines out of 1,024. The other effects vary slowly compared to the dislocation lattice, and should be approximated reasonably well by a power series in $\mathbf{r}$. To eliminate them, we subtracted a 4th-order polynomial fit from each component of $\nabla\mathbf{u}(\mathbf{r})$.

The smoothing length L also plays an important role. We used Gaussian smoothing (i.e. the smoothing function $S(\mathbf{r})$ was $N_0 e^{-r^2/2L^2}$). For Fig. 2, we used L = 2 nm; for the mask-averaged strain (Figs. 4 and 5) we used L = 1.5 nm.

Supplementary Note II

To spatially filter the dI/dV data in a way that captures the variation of a single type of strain, while suppressing the variation of its complement, we devised spatial masking functions $M(\mathbf{r})$ which share the quasi-periodicity of the topographic superlattice. (Here $M(\mathbf{r}) \in [0,1]$, and the object to be Fourier-transformed is $G_M(\mathbf{r}) \equiv M(\mathbf{r})dI/dV(\mathbf{r})$. To suppress high-wavevector noise $M(\mathbf{r})$ is given a smooth roll-off.)

As described in the main text, we used two basic species of masking function: 1D, linear masks following the direction of one set of topographic troughs; and 2D, quasi-circular masks which pick out a certain part of the superlattice unit cell. In Supplementary Fig. 1, the former is represented by masks III and IV (which capture variation of u_{xx} and u_{yy} respectively), while the latter is represented by masks I and II (which capture respectively C and U). If we define the superlattice unit cell so that two troughs intersect at (0,0), and the "anti-trough" intersection is at (0.5,0.5), then mask II can be obtained by translating mask I rigidly through (0.5,0).

To define this coordinate system numerically, we located each trough (by finding the local maximum of the smoothed second derivative of the topography, averaged over the direction nominally parallel to it)

and assigned it a sequential index, making the rectilinear, quasi-Cartesian grid shown in Supp. Figure 2b. This grid defines a 2-D coordinate system which was used to formulate the $M(\mathbf{r})$.

All $M(\mathbf{r})$used are periodic with respect to this grid, and can be defined in terms of the distance between each $\mathbf{r}$and a certain point, or locus of points, within the dislocation-lattice unit cell. For each pixel $\mathbf{r}$, we define the dislocation-grid distance between $\mathbf{r}$ and the nearest point whose dislocation-grid coordinates modulo 1 are (μ, ν). This is

$$d(\mathbf{r}) = \sqrt{((a-\mu) - Round(a-\mu))^2 + ((b-\nu) - Round(b-\nu))^2},$$

where (a, b) are the dislocation-grid coordinates of $\mathbf{r}$ and the $Round$ function rounds its argument to the nearest integer. (Clearly, $d(\mathbf{r}) \in [0, \sqrt{2}]$.)

For the one-dimensional masks we are interested only in one coordinate or the other:

$$d_a(\mathbf{r}) = |(a-\mu) - Round(a-\mu)|,$$

$$d_b(\mathbf{r}) = |(b-\nu) - Round(b-\nu)|;$$

then $d(\mathbf{r}) = \sqrt{{d_a}^2 + {d_b}^2}$. For the case $\mu, \nu = 0$ these distances are shown in **Supp. Fig. 2 (d)-(f)** respectively. For each type of strain the mask was a well-known monotonic function of the corresponding distance; for the one-dimensional masks we used Gaussians:

$$M_{xx}(\mathbf{r}) = e^{-{d_a}^2/\lambda^2}, M_{yy}(\mathbf{r}) = \ e^{-{d_b}^2/\lambda^2}$$

where the subscripts denote the type of strain we want to vary, and λ is chosen so that the mask should include a certain fraction of the total spectral weight, 1/3rd for both (i.e., $\int d\mathbf{r}M(\mathbf{r}) = 1/3$). We used $\lambda = 0.186$, found by binary search.

For the 2-D masks we used the Fermi function:

$$M(\mathbf{r}) = 1/(1 + e^{(d(\mathbf{r})-\varphi)/T})$$

with $T = 0.05$ and $\varphi = 0.268$, designed so that $\int d\mathbf{r}M(\mathbf{r}) = 1/4$.

The masks were translated by varying (μ, ν). For M_{xx} and M_{yy}, μ and ν respectively were swept from 0 to -2 in the supplementary movies; from 0 to -1/2 in Fig. 4. For isotropic compression (μ, ν) was swept from (0,0) to (-2,-2) in the movies; for uniaxial stretch the sweep was from (0, 0.5) to (-2,-1.5).

Actually, for all four types of masks the sweeps could be expressed as

$$(\mu, \nu) = (\mu_0, \nu_0) + \eta(-1, -1)$$

where the "mask position variable" η goes from 0 to 2, and is the abscissa of the graphs in **Supp. Fig. 5(a)-(e)**; it also appears in the y-axis of **Supp. Fig. 3**. Because of the periodicity the range 1 to 2 is equivalent to 0 to 1; it was included for aesthetic reasons in **Supp. Fig. 5**, and in order to make the movies more watchable.

We note here that because the masked dI/dV was largely the same for adjacent values of η ($\mathrm{d}\eta = 0.03$ for 64 points between 0 and 2) the scatter plots in **Supp. Fig. 5** do not represent as many independent measurements of $\Delta\mathbf{Q_1}$ as their number of points might be taken to imply. We guess that the number of *truly* independent measurements of $\Delta\mathbf{Q_1}$ is the reciprocal of the fraction of spectral weight contained in the mask: 3 for u_{xx} and u_{yy}; 4 for C and U. Since the overlap between different masking functions is finite by design, these three or four inherently different measurements cannot be strictly separated, as in Ref. [3]. Their number, however, is sufficient for fitting to a straight line.

Supplementary Note III

Because $\Delta\mathbf{Q_1}$ was the shift of a rather broad peak, smeared out by mask-induced ringing, the quality and consistency of the peak-fitting procedure was important. Before fitting for $\mathbf{Q_1}$ the Fourier transforms of $G_M(\mathbf{r})$ were subjected to the following: (1) Reflection-symmetrization about one of the basic reciprocal lattice vectors ($\hat{\mathbf{x}}$, but since the magnitude of the FFT is inversion-symmetric using $\hat{\mathbf{y}}$ would have given the same result); (2) k-space smoothing on a length scale sufficient to wash out the ringing; we used Gaussian smoothing ($N_0 e^{-r^2/2L^2}$ with $L = 0.048$Å^{-1} = 10 momentum-space pixels). Then (3) we took the Laplacian in k-space (multiplied by -1) to accentuate the peak features.

Step (1) was applied to Fourier transforms in **Fig. 3**. Its effect is to make the QPI feature mirror-symmetric about the line bisecting it, so that each half-QPI peak makes it possible for each half-peak in **Fig. 3(b)-(e).**

The results of the three steps, as applied to the Fourier transform of the unmasked dI/dV (**Supp. Fig. 4(a)**), are shown in **Supp. Fig. 4 (b)-(d)**. The thus-processed FFTs of the masked dI/dV at $\eta = 0$ and 0.5, the same as **Fig. 3 (b)-(e)** of the main text, are shown in panels **Supp. Fig. 4 (e)-(h)**. One of the consequences of the processing is that the inner scattering peak, denoted $\mathbf{Q}_1'$ in **Supp. Fig. 4 (d)**, is plainly visible in the FFTs of the masked data. This vector represents intra-pocket scattering along the long axes of the ellipses, and should be equally indicative with $\mathbf{Q}_1$ of the strain-induced shifts in the Dirac cones predicted by equations (1) and (2). The geometry of the constant energy contour (**Supp. Fig. 4(d)** inset) shows that the sum $\mathbf{Q}_1'{}_{x,y}$ + $\mathbf{Q}_{1x,y}$ = one reciprocal lattice vector, and indeed we see in **Supp. Fig. 4 (e)-(h)** that a significant shift of $\mathbf{Q}_{1x,y}$ of is always associated with a roughly equal shift of $\mathbf{Q}_1'{}_{x,y}$ in the opposite direction.

To actually extract $\mathbf{Q}_{1x,y}$ we took line cuts in the fully-processed FFT in the $\hat{\mathbf{x}}$ and $\hat{\mathbf{y}}$ directions (from the origin), and fit the peak to an inverted parabola; the fitting window had a half-width of 0.04 Å^{-1}, and its position shifted with η to keep the peak centered.

Supplementary Note IV

The variation in $\mathbf{Q}_{1x,y}$ and the average strain $\overline{u_{ij}}$ clearly depends on the specific functional form of $M(\mathbf{r})$. To examine this dependence, we repeated the fitting and masking procedure for compressive strain with several different functions. The results are shown in **Supp. Figs. 5** and **6**. In **Supp. Fig. 5** one finds that tighter masking functions lead to greater variation in both the average strain and the $\mathbf{\Delta Q}$'s. Although there is some trend observed, with looser masks associated with slightly steeper slopes, for all five masks the slopes were within 10% of their mean.

Supplementary Note V

SnTe belongs to space group 225. Yet, when strain is applied to one of the axis (a~=b), the space group of SnTe changes into SG 71 (**Supp. Fig. 7(a,b)**). To describe SnTe under strain in one axis, we build a simple Tight-binding (TB) model under Slater and Koster's method. For simplicity, we only consider hoppings between P orbitals the nearest Sn-Te, Sn-Sn, Te-Te, which are labeled as V_{Sn-Te}, V_{Sn-Sn} and V_{Te-Te}. For each hopping, we could decompose it into σ and π bounds:

$$V_{AB}^{\sigma} = \alpha_{AB}^{\sigma} * e^{-\beta r_{AB}} * \sqrt{rd_A * rd_B}$$
$$V_{AB}^{\pi} = \alpha_{AB}^{\pi} * e^{-\beta r_{AB}} * \sqrt{rd_A * rd_B}$$

Where r_{AB} is distance between A and B atoms, α_{AB}^{σ} and α_{AB}^{π} decide the strength of related hoppings, β decides the decay rate of this hopping with distance, rd_A and rd_B are constants related to each atom. The onsite energy of each atom is labeled as m, and the effects of spin-orbit coupling (SOC) are described by λ. Using the parameters listed in **Supp. Table 2**, the above TB model roughly reproduces the results from DFT, as shown in Fig. S7(c).

In this section, we show the surface states and QPI patterns from the above TB model of SnTe after apply external strain along the x direction. **Supp. Fig. 8(a-c)** shows the E-K dispersions of surface Dirac Fermions on the x and y directions under different strains. One could see that decreasing (increasing) the x-axis will increase (decrease) the energy dispersions along y-direction. As a result, the size of Fermi circles enclosing y-axis will increase with the decreasing of length of x-axis. This effect could be clearly observed in QPI patterns, as shown in **Supp. Fig. 8(d,e)**, which is consistent with experimental measurements.

References

1. Hÿtch, M. J., Snoeck, E. & Kilaas, R. Quantitative measurement of displacement and strain fields from HREM micrographs. *Ultramicroscopy* **74,** 131–146 (1998).

2. Lawler, M. J. *et al.* Intra-unit-cell electronic nematicity of the high-Tc copper-oxide pseudogap states. *Nature* **466,** 347–351 (2010).

3. Zeljkovic, I. *et al.* Strain engineering Dirac surface states in heteroepitaxial topological crystalline insulator thin films. *Nat. Nanotechnol.* **10,** 849–853 (2015).
