# Supplementary material for: Interplay of orbital effects and nanoscale strain in topological crystalline insulators
Source: Nat Commun. 2018 Apr 19;9:1550. doi: 10.1038/s41467-018-03887-5 (PMC5908802; doi:10.1038/s41467-018-03887-5)
Supplement: Supplementary file 2 — Description of Additional Supplementary Files [file 41467_2018_3887_MOESM2_ESM.pdf]

**Description of Additional Supplementary Files**

File Name: Supplementary Movie 1
Description: Movie showing effect of compressive/tensile strain Dirac surface states

File Name: Supplementary Movie 2
Description: Movie showing effect of uniaxial strain Dirac surface states
